# Orthoplastic management of delayed sternal osteomyelitis and non-union: a retrospective case series applying fracture-related infection principles

**DOI:** 10.1007/s00264-026-06916-x

**Published:** 2026-06-22

**Authors:** Katie Hutchinson, Jamie Banks, Amber Arnold, Jonathan Lohn, Alex Trompeter

**Affiliations:** 1 St George’s University Hospitals NHS Foundation Trust, London, UK; 2 Chelsea and Westminster Hospital NHS Foundation Trust, London, UK; 3 St George’s, University of London, London, UK

**Keywords:** Bone infection, Osteomyelitis, Fracture related infection, Orthoplastic

## Abstract

**Background:**

Sternal osteomyelitis (OM), particularly in the context of late-presenting non-union following cardiothoracic surgery, remains a poorly understood and inconsistently managed condition. Delayed presentations involve established infection, sternal instability, and biofilm formation, requiring a multidisciplinary approach. BOAST guidelines for fracture-related infection (FRI) recommend a combined orthoplastic approach to manage these complex cases.

**Methods:**

This retrospective case series identified all patients referred to a specialist bone infection unit with delayed onset sternal OM from March 2015 to March 2025. All patients were managed through a multidisciplinary pathway involving cardiothoracic surgery, orthopaedics, plastic surgery, and microbiology. Each case involved planning through a bone infection MDT, radical debridement, multiple deep tissue sampling, removal of infected foreign material, skeletal stabilisation where indicated, definitive soft tissue reconstruction, and culture-directed antimicrobial therapy. Clinical outcomes, microbiological findings, and recurrence were assessed.

**Results:**

Fourteen patients were included (mean age 63·7 years), all with significant comorbidity burden. The interval from index cardiac surgery to definitive debridement ranged from 25 to 3,251 days. Eleven patients underwent single-stage debridement with immediate reconstruction: three required staged procedures. The most commonly isolated organisms were *Staphylococcus epidermidis* (7/14), *Cutibacterium acnes* (5/14), and *Staphylococcus aureus* (4/14); mixed infections were frequent. Mean length of stay following single-stage procedures was 10·5 days. During a mean follow-up of 410 days, one patient experienced recurrence requiring further surgery.

**Conclusion:**

This case series demonstrates that delayed sternal OM and non-union can be effectively managed through an orthoplastic approach aligned with FRI principles. Isolated soft tissue coverage, which has historically been the default management, fails to address the underlying pathology. Approaches focused solely on soft tissue reconstruction risk, persistent infection, and instability. This study supports the application of fracture-related infection principles to delayed sternal osteomyelitis, emphasising radical debridement, hardware removal, multidisciplinary decision-making, and definitive reconstruction as key components of successful treatment.

## Introduction

Sternal osteomyelitis (OM) is an uncommon but serious complication following midline sternotomy, associated with considerable morbidity [1]. Deep sternal wound infection (DSWI) and sternal osteomyelitis are frequently conflated within the literature despite representing distinct clinical entities. DSWI encompasses a spectrum of postoperative infections involving the sternum, mediastinum and surrounding soft tissues, typically presenting in the early postoperative period. In contrast, delayed sternal osteomyelitis is characterised by established bony infection, often associated with chronic sinus formation, retained hardware, biofilm development and occasionally sternal non-union [2]. These distinctions have important implications for treatment and surgical planning.

The management of implant-related infection in orthopaedics has evolved considerably through the development of fracture-related infection (FRI) principles. Delayed sternal osteomyelitis shares many features with FRI, including chronic infection, retained fixation material, biofilm formation, osseous destruction, and mechanical instability. Consequently, established FRI treatment principles, including multidisciplinary planning, radical debridement, implant removal, deep microbiological sampling, dead space management, skeletal stabilisation, and culture-directed antimicrobial therapy, may provide a useful framework for managing these complex infections. However, the application of these principles to the sternum remains largely unexplored within the literature.

Traditional management has often focused on soft tissue reconstruction alone, without adequately addressing underlying osteomyelitis, retained foreign material or instability. This can result in persistent infection, mechanical instability and suboptimal outcomes. The management of implant related infection in orthopaedics has evolved considerably through the development of fracture related infection (FRI) principles. Delayed sternal osteomyelitis shares many features with FRI, including chronic infection, retained fixation material, biofilm formation, osseous destruction and mechanical instability. FRI is defined by confirmatory and suggestive diagnostic criteria; confirmatory criteria include purulent drainage, a sinus tract or fistula communicating with bone or implant, and positive microbiology or histopathology, whilst suggestive criteria include clinical signs, elevated inflammatory markers and supportive radiological findings. Consequently, established FRI principles, including multidisciplinary planning, radical debridement, implant removal, deep microbiological sampling, dead space management, skeletal stabilisation and culture directed antimicrobial therapy, may provide a useful framework for managing these complex infections. However, their application to the sternum remains largely unexplored.

Recent guidelines have provided a more structured approach to musculoskeletal infection, particularly in managing fracture-related infection (FRI) [3]. Despite the clinical importance of sternal OM, the literature remains sparse and fragmented, with no current consensus on optimal treatment strategies. International guidance and national standards, including the recent British Orthopaedic Association Standards for Trauma (BOAST) on FRI, emphasise the importance of multidisciplinary collaboration and planning, thorough debridement, skeletal stabilization, soft tissue coverage, and targeted antimicrobial therapy [4]. Management should be individualised and led by experienced, multidisciplinary teams incorporating microbiology, orthopaedic and plastic surgery expertise.

International guidance and national standards, including the British Orthopaedic Association Standards for Trauma (BOAST) on FRI, emphasise multidisciplinary collaboration, thorough debridement, skeletal stabilisation, soft tissue reconstruction and targeted antimicrobial therapy.^4^ Despite the clinical importance of sternal OM, the literature remains sparse, with no clear consensus on optimal management.

The aim of this study was to evaluate a consecutive series of patients with delayed sternal osteomyelitis managed using an orthoplastic approach based on contemporary fracture related infection principles, including BOAST guidance.

## Methods

A retrospective analysis of a prospective bone infection database was performed. A consecutive series of patients referred to a single specialist major bone infection unit in the UK between March 2015 and March 2025 were included.

Data collected included patient demographics, comorbidities, initial cardiac procedure, time from index surgery to infection, microbiological findings, imaging results, operative details, antibiotic regimens, complications, and clinical outcomes.

All patients were assessed and managed through a structured MDT pathway involving referral from cardiothoracic services, specialist imaging, multidisciplinary review, definitive orthoplastic debridement, microbiological analysis, reconstruction, culture-directed antimicrobial therapy, and longitudinal follow-up.

### Inclusion criteria

- *A history of median sternotomy for cardiac surgery.*
- *Clinical and/or radiological evidence of sternal OM, defined by the presence of a draining sinus, deep-seated infection, or imaging-confirmed osteomyelitis.*
- *Management involving combined orthoplastic microbiological approach.*

### Exclusion criteria

- *Patients with acute dehiscence post-surgery without evidence of osteomyelitis, or incomplete documentation.*
- *Non-operative management.*

Surgical management adhered to FRI principles, including multiple deep separate surgical samples, thorough debridement of infected and necrotic tissue, removal of any retained metalwork, rigid sternal fixation where needed, and definitive soft tissue reconstruction. Where bone defects were present local antimicrobials were used. Empirical antibiotic therapy was tailored to the identified pathogens and continued postoperatively, with duration guided by clinical, biochemical, and radiological response. All cases were reviewed at a dedicated multidisciplinary infection team (MDT) meeting to determine ongoing management and follow-up.

## Results

Between March 2015 and March 2025, fourteen patients met the inclusion criteria. Of these, five were female and nine were male. The age range was 40 to 84 years, with a mean age of 63.71 years (standard deviation [SD] 12.9). All patients had a history of initial sternotomy closure with steel wiring. All patients were ASA4 due to advanced cardiac disease; one patient had only two major comorbidities (valvular heart disease and a pacemaker), while the remainder had three or more, with the maximum being nine comorbidities in two patients (Table 1).

**Table 1:** Prevalence of baseline comorbidities among patients with confirmed sternal osteomyelitis

Comorbidity	Prevalence. (*n*)
*Coronary artery disease*	11
*Hypertension*	7
*Hyperlipidaemia*	7
*Respiratory disease (asthma, chronic obstructive pulmonary disease, or bronchiectasis)*	6
*Diabetes mellitus*	6
*Valvular heart disease*	4
*Solid organ malignancy*	2
*Connective tissue disease*	2
*Heart failure*	1
*Pressure ulcer*	1
*Peripheral vascular disease*	1

Regarding primary cardiac surgery, ten patients underwent coronary artery bypass grafting, while the remaining four underwent valvular procedures (three aortic and one mitral). The interval between cardiac surgery and first orthoplastic debridement varied considerably, reflecting both acute and chronic presentations of sternal osteomyelitis. This interval ranged from 25 to 3,251 days, with a mean of 872 days (SD 1,145). Four patients had previously undergone debridement by cardiothoracic teams, including removal of sternal wires.

**Fig. 1:**
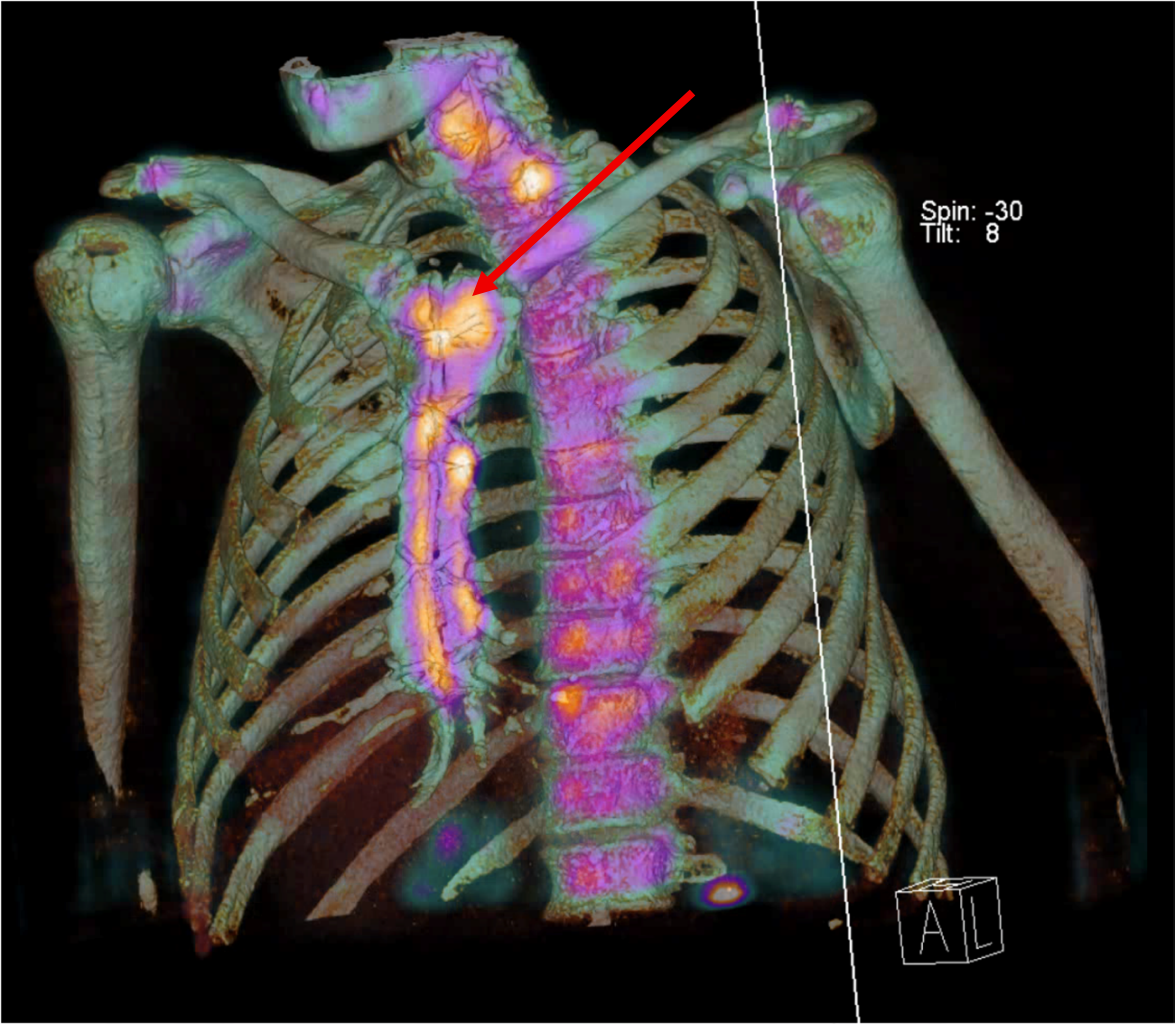
3D reformat of a SPECT CT demonstrating high signal within the sternum

**Fig. 2:**
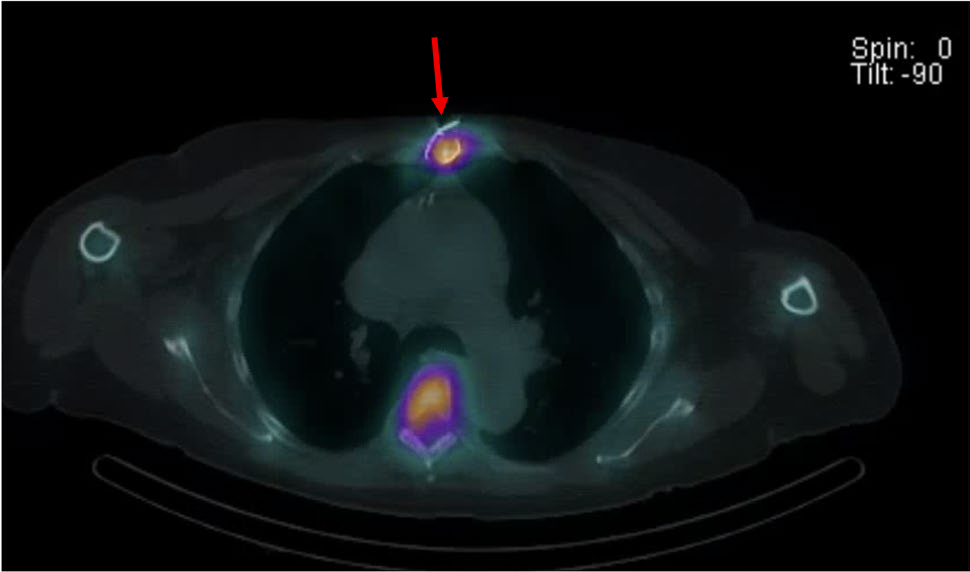
Axial SPECT CT image demonstrating high signal within the sternum

**Fig. 3:**
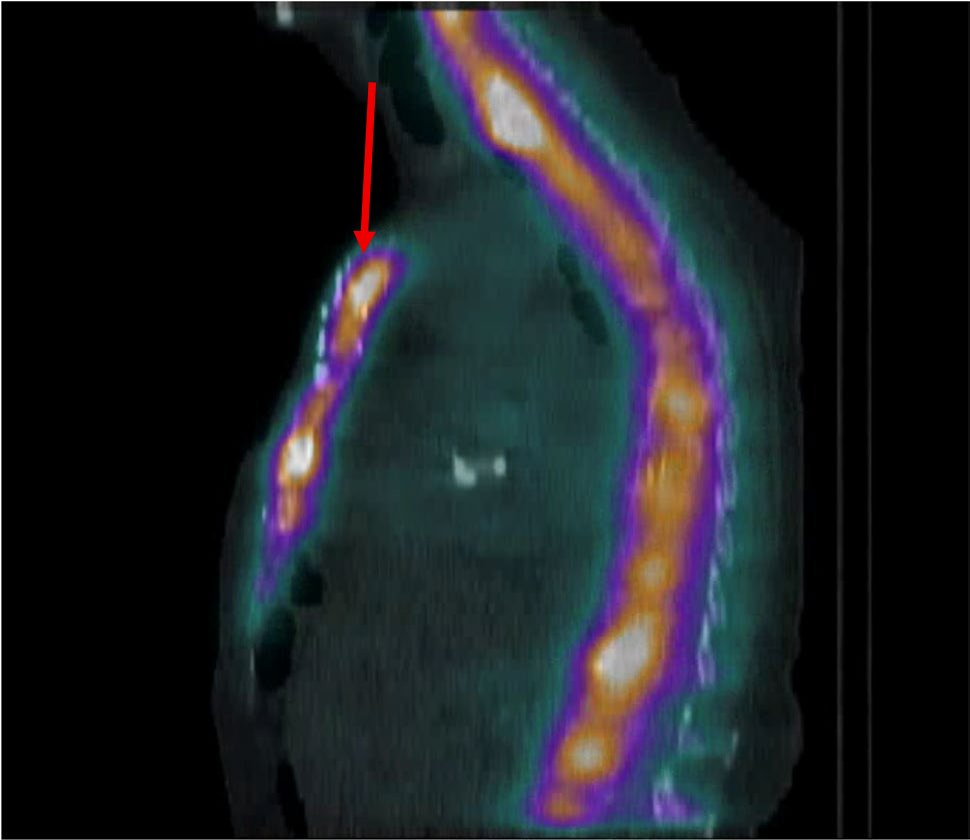
Sagittal SPECT CT image demonstrating high signal within the sternum

All fourteen patients met microbiological and clinical definitions of infection, fulfilling confirmatory criteria according to the AO/EBJIS fracture-related infection diagnostic framework, and all demonstrated radiological features consistent with osteomyelitis on SPECT–CT imaging (Figs. 1, 2 and 3). Two had non-union in conjunction with the infection. Following transfer of care from the cardiothoracic team, eleven patients underwent single-stage orthoplastic debridement and soft tissue coverage. Of these, two chronic cases (> 2,900 days post-index procedure), were managed via dead space eradication, in which local antimicrobials, either Cerament (BONESUPPORT AB, Lund, Sweden) or vancomycin powder, were applied.

Three cases required staged procedures following transfer of care, with at least two serial debridements by orthoplastic teams, prior to a separate final definitive soft tissue reconstruction; in these cases, the interval from first orthoplastic debridement to coverage ranged from seven to fourteen days.

Soft tissue coverage was achieved by direct closure in two patients. The remaining twelve underwent reconstruction using pectoralis major muscle flaps, including four bilateral advancements and eight unilateral advancements. One of these unilateral advancements was combined with a rectus abdominis turnover flap for inferior wound coverage. One patient required iliac crest bone grafting and plate fixation, performed concurrently with the initial debridement as a single-stage procedure for a painful mobile non-union.

Post-operative inpatient stay ranged from six to 101 days (mean 20.8 days, SD 28.4). Among patients who underwent single-stage coverage, hospital stay ranged from six to 20 days, with a mean of 10.5 days.

Microbiological findings were diverse. In cases where it was recorded, there were between three and ten microbiology samples taken intraoperatively including both tissue samples and sonication samples from removed metalwork. The most frequently isolated organism was *Staphylococcus epidermidis* (*n* = 7), followed by *Cutibacterium Acnes* (formerly *Propionibacterium acnes*) (*n* = 5) and *Staphylococcus aureus* (*n* = 4), shown in Table 2. Eight cases demonstrated mixed flora, and two patients had sterile cultures, one of which had negative further PCR analysis; these individuals were managed empirically with broad-spectrum antibiotics post operatively.

**Table 2:** Incidence of bacterial pathogens isolated in patients with sternal osteomyelitis

Organism	Incidence (*n*)
*Staphylococcus epidermis*	7
*Cutibacterium acnes*	5
*Staphylococcus aureus*	4
*Corynebacterium* species	2
*Pseudomonas aeruginosa*	1
*Pseudomonas stutzeri*	1
*Staphylococcus haemolyticus*	1
*Enterobacter cloacae*	1
*Staphylococcus hominis*	1

In mixed flora cases after further analysis, *s. aureus* was deemed to be the significant organism in one case and *p. acnes* in two cases.

Four patients were documented to have had an antibiotic holiday defined as two weeks off antibiotics pre sampling or to be antibiotic naive at time of operation. Conversely, five patients were documented to have continued oral or intravenous antibiotics in the immediate pre-operative phase. There was no preoperative antibiotic data for the remaining patients.

Post-operative antibiotic choice and course lengths varied from just 48 h to 12 weeks with varying regimes of intravenous and oral combinations. Course and choice were directed by a microbiology team with a specialist interest in bone infection.

Follow-up, recorded through either orthoplastic or cardiothoracic outpatient services, ranged from 34 days to 1,633 days, with a mean duration of 410 days (SD 508). There was a single documented case of recurrence in a patient with nine major comorbidities who underwent definitive closure, with no recurrence at follow-up six months later. One patient was palliated and died 45 days post definitive coverage.

## Discussion

Sternal OM remains a rare but potentially catastrophic complication following midline sternotomy, with a significant mortality rate, often presenting in a delayed fashion with non-specific clinical signs [5]. The insidious nature of this infection highlights the importance of timely referral to a multidisciplinary team, including plastic, microbiology and orthopaedic surgeons, to ensure appropriate intervention [6]. Sternal OM, especially in its chronic form, continues to be poorly understood and inadequately addressed in the literature, likely due to the lack of robust, evidence-based guidelines informing management.

Historically, the treatment of post-sternotomy infections has typically involved superficial wound debridement and empirical antibiotic therapy, with limited attention to the bony involvement that underpins chronic sternal OM [7]. This approach was insufficient for achieving long-term infection control and preventing recurrence. Modern FRI treatment strategies emphasise thorough debridement of soft tissue and bone, targeted antimicrobial therapy, and definitive skeletal stabilisation [4]. The management of sternal OM necessitates a comprehensive approach that includes early and aggressive surgical intervention to eradicate infection and restore chest wall integrity. Integrating orthoplastic principles, with a focus on multidisciplinary collaboration, is pivotal in achieving optimal outcomes in these complex cases [8].

A key finding of this series is that successful treatment required addressing both the infectious and mechanical aspects of disease. Historically, delayed sternal infection has often been managed with soft tissue reconstruction alone, without adequately addressing underlying osteomyelitis, retained hardware, or instability. In contrast, the orthoplastic approach combines radical debridement, hardware removal, skeletal reconstruction where required, and definitive soft tissue coverage within a multidisciplinary pathway. By addressing chronic infection, biofilm formation, and non-union concurrently, this strategy may account for the favourable outcomes observed.

The cornerstone of effective management in sternal OM is a methodical and evidence-based approach, rather than initiating empirical antibiotic therapy or performing indiscriminate en bloc resections. The recommended strategy involves thorough surgical debridement with the collection of deep bone and soft tissue specimens for microbiological analysis prior to the commencement of antimicrobial treatment [9]. Where possible, an antibiotic holiday, where relevant, is enforced for several weeks prior to surgical intervention. Intravenous antibiotics are initially broad spectrum based on local best guess sensitivities given after sampling (empirical Vancomycin and Gentamicin in our institution), moving on to culture-directed therapy, initiated once representative microbiology results have been obtained, ensuring targeted and effective therapy.

Complete removal of all retained foreign material from the infected site is crucial [10]. Retention of hardware, such as sternal wires or plates, perpetuates infection and significantly reduces the likelihood of successful outcomes. Pharmacological infection suppression while colonised implants remain in situ is rarely successful and often result in recurrence. Contemporary definitive management must prioritise eradication of infection through radical debridement, removal of all foreign material, and timely soft tissue reconstruction.

The collection of multiple deep tissue samples using separate sterile instruments for each is of paramount importance, enabling reliable differentiation between true infection and contamination [11]. The consistent isolation of a pathogen across the majority or entirety of samples (e.g. 4/5 or 5/5) provides strong microbiological evidence of infection, whereas growth in only a single sample is more likely indicative of contamination. Such clarity is essential for guiding appropriate antimicrobial therapy, determining its duration, and informing the timing and planning of reconstructive surgery. Strict adherence to meticulous sampling protocols should therefore be regarded as a fundamental aspect of multidisciplinary infection management.

The current body of literature surrounding the management of sternal osteomyelitis remains limited in both scope and quality. Most publications are composed of retrospective case series, single-centre experiences, or narrative reviews, which restrict the generalisability of findings. There is a notable lack of prospective studies, randomised controlled trials, or standardised treatment protocols specific to sternal OM, in contrasts to the growing evidence base for long bone and periprosthetic infections. The current literature suffers from inconsistent terminology, with many studies conflating deep sternal wound infection (DSWI) with established OM, despite differences in pathophysiology and treatment requirements. This ambiguity complicates efforts to determine true incidence, risk stratification, and optimal timing of intervention.

There is considerable heterogeneity in surgical approaches in the literature, ranging from limited debridement to subtotal sternectomy, with varying use of skeletal stabilisation techniques [12]. Similarly, soft tissue reconstruction strategies differ widely, and there is minimal consensus on the most appropriate flap choice or timing of reconstruction. It is our view that total sternal soft tissue coverage is often unnecessary and the morbidity of coverage of the lower quarter of the sternum which usually necessitates use of an abdominal flap can often be avoided with the integrated multidisciplinary involvement that we advocate. In addition, our series clearly demonstrates that single stage surgery is both safe and effective in the management of complex sternal infection. The literature rarely reflects the critical role of multidisciplinary team (MDT) working, particularly the integration of orthopaedic, plastic surgical, cardiothoracic, and microbiological expertise. Where MDT care is described, it is often anecdotal rather than being evaluated as a determinant of outcome. Current literature lacks methodological rigour, standardisation, and prospective data. High-quality, collaborative research is needed to establish validated treatment algorithms, incorporate patient-reported outcomes, and define the role of newer surgical and antimicrobial techniques.

This study has several limitations. First, the retrospective design introduces the potential for selection and reporting bias. Second, the cohort size is relatively small, reflecting the rarity of delayed sternal osteomyelitis requiring specialist orthoplastic management. Third, follow-up duration varied considerably between patients, limiting assessment of very long-term recurrence rates. Finally, patient-reported outcome measures were not routinely collected and therefore functional recovery and patient satisfaction could not be formally evaluated. Despite these limitations, this series represents one of the largest dedicated orthoplastic cohorts reported for delayed sternal osteomyelitis and provides valuable data on a poorly described condition.

Despite the limitations inherent in a single-centred, retrospective study, this study offers a valuable insight into managing a poorly understood condition. It reinforces the necessity of a holistic, coordinated approach that addresses the local pathology and the patient’s broader physiological and psychosocial context. Future research should focus on prospective multi-centre studies with longer-term follow-up, standardised outcome reporting, and incorporation of patient-reported outcome measures. Such studies would help validate the reproducibility of these findings and further define the role of fracture-related infection principles in managing delayed sternal osteomyelitis and non-union.

## Conclusion

Sternal osteomyelitis remains a rare but serious complication with significant morbidity. Traditional approaches to its management are often fragmented and inadequate, failing to address the disease's bony and infectious components. This study series highlights the importance of adopting a structured, multidisciplinary approach.

The application of contemporary fracture related infection principles, including early diagnosis, radical surgical debridement, skeletal stabilisation and culture directed antimicrobial therapy, may lead to improved clinical outcomes. Integrating ortho-plastic techniques and microbiological oversight ensures comprehensive care, reduces the risk of recurrence, and promotes timely recovery. These findings support the growing consensus that complex sternal infections require coordinated management within specialised centres, guided by national standards and multidisciplinary expertise.

## Data Availability

No datasets were generated or analysed during the current study.
